# Characterizing Hypertension Specialist Care in Canada: A National Survey

**DOI:** 10.1016/j.cjco.2023.08.014

**Published:** 2023-09-10

**Authors:** Samantha Lui, Lisa Dubrofsky, Nadia A. Khan, Sheldon W. Tobe, Jessica Huynh, Laura Kuyper, Anna Mathew, Syed Amin, Ernesto L. Schiffrin, Paula Harvey, Alexander A. Leung, Marcel Ruzicka, Birinder Mangat, David Reid, John Floras, Jesse Bittman, Lauren Garbutt, Branko Braam, Rita Suri, Fady Hannah-Shmouni, Ally Prebtani, Sebastien Savard, Thomas E. MacMillan, Terrence D. Ruddy, Michel Vallee, Apoorva Bollu, Alexander Logan, Raj Padwal, Jennifer Ringrose

**Affiliations:** a Department of Medicine, University of Alberta, Edmonton, Alberta, Canada; b Department of Medicine, Women’s College Hospital, Toronto, Ontario, Canada, Division of Nephrology, Department of Medicine, University of Toronto, Toronto, Ontario, Canada; c Department of Medicine, University of British Columbia, Vancouver, British Columbia, Canada; d Division of Nephrology Sunnybrook Health Sciences Centre, University of Toronto, Toronto and Northern Ontario School of Medicine, Sudbury, Ontario, Canada; e Department of General Internal Medicine, Faculty of Health Sciences, McMaster University, Hamilton, Ontario, Canada; f Division of General Internal Medicine, University of British Columbia, Vancouver, British Columbia, Canada; g Division of Nephrology, Department of Medicine, St. Joseph Healthcare Hamilton, Hamilton, Ontario, Canada; h Division of Nephrology, Saskatchewan Health Authority, Regina, Saskatchewan, Canada; i Department of Medicine, Lady Davis Institute for Medical Research, Sir Mortimer B. Davis-Jewish General Hospital, McGill University, Montreal, Quebec, Canada; j Division of Cardiology, Department of Medicine and Women’s College Research Institute, Women’s College Hospital, University of Toronto, Toronto, Ontario, Canada; k Departments of Medicine and Community Health Sciences, Cumming School of Medicine, University of Calgary, Calgary, Alberta, Canada; l Division of Nephrology, University of Ottawa, Ottawa, Ontario, Canada; m Dvision of Nephrology, University of Saskatchewan, Saskatoon, Saskatchewan, Canada; n University Health Network and Sinai Health Department of Medicine, University of Toronto, Toronto, Ontario, Canada; o Division of Community Internal Medicine, University of British Columbia, Vancouver, British Columbia, Canada; p Division of Endocrinology, University of Manitoba, Winnipeg, Manitoba, Canada; q Department of Medicine, Division of Nephrology, University of Alberta, Edmonton, Alberta, Canada; r Division of Nephrology, Department of Medicine, McGill University Health Centre, Montreal, Quebec, Canada, Research Institute of the McGill University Health Centre, Montreal, Quebec, Canada; s Division of Endocrinology, University of British Columbia, Vancouver, British Columba, Canada, Eunice Kennedy Shriver National Institute of Child Health and Human Development (NICHD), National Institutes of Health (NIH), Bethesda, Maryland, USA; t Division of Endocrinology & Metabolism, Department of Medicine, McMaster University, Hamilton, Ontario, Canada; u Department of Medicine, Universite Laval, Hotel-Dieu de Quebec, Quebec City, Quebec, Canada; v Department of Medicine, Division of General Internal Medicine, University of Toronto, Toronto, Ontario, Canada; w Division of Cardiology, University of Ottawa Heart Institute, Ottawa, Ontario, Canada; x Faculté de Médecine, Université de Montréal, Montreal, Quebec, Canada; y Department of Medicine, University of Toronto, Toronto, Ontario, Canada

## Abstract

**Background:**

The hypertension specialist often receives referrals of patients with young-onset, severe, difficult-to-control hypertension, patients with hypertensive emergencies, and patients with secondary causes of hypertension. Specialist hypertension care compliments primary care for these complex patients and contributes to an overall hypertension control strategy. The objective of this study was to characterize hypertension centres and the practice patterns of Canadian hypertension specialists.

**Methods:**

Adult hypertension specialists across Canada were surveyed to describe hypertension centres and specialist practice in Canada, including the following: the patient population managed by hypertension specialists; details on how care is provided; practice pattern variations; and differences in access to specialized hypertension resources across the country.

**Results:**

The survey response rate was 73.5% from 25 hypertension centres. Most respondents were nephrologists and general internal medicine specialists. Hypertension centres saw between 50 and 2500 patients yearly. A mean of 17% (± 15%) of patients were referred from the emergency department and a mean of 52% (± 24%) were referred from primary care. Most centres had access to specialized testing (adrenal vein sampling, level 1 sleep studies, autonomic testing) and advanced therapies for resistant hypertension (renal denervation). Considerable heterogeneity was present in the target blood pressure in young people with low cardiovascular risk and in the diagnostic algorithms for investigating secondary causes of hypertension.

**Conclusions:**

These results summarize the current state of hypertension specialist care and highlight opportunities for further collaboration among hypertension specialists, including standardization of the approach to specialist care for patients with hypertension.

## Résumé

**Contexte:**

Le spécialiste de l’hypertension reçoit souvent des patients orientés pour une hypertension sévère, d’apparition précoce et difficile à maîtriser, pour une urgence hypertensive ou pour des causes secondaires de l’hypertension. Les soins spécialisés de l’hypertension complètent les soins primaires pour ces cas complexes et font partie d’une stratégie globale de maîtrise de l’hypertension. Cette étude avait pour objectif de caractériser les centres de traitement de l’hypertension et les habitudes de pratique des spécialistes canadiens qui traitent l’hypertension.

**Méthodologie:**

Un sondage a été mené auprès de spécialistes de l’hypertension adulte de l’ensemble du Canada afin de décrire les centres de traitement de l’hypertension et la pratique des spécialistes au Canada, notamment les éléments suivants : la population de patients prise en charge par des spécialistes de l’hypertension, les renseignements sur la façon dont les soins sont prodigués, les variations dans les habitudes de pratique ainsi que les différences relatives à l’accès aux ressources spécialisées en hypertension à l’échelle du pays.

**Résultats:**

Le taux de réponse au sondage a été de 73,5 % dans 25 centres de l’hypertension. La plupart des répondants étaient des néphrologues et des spécialistes en médecine interne générale. Les centres de l’hypertension recevaient entre 50 et 2500 patients par année. En moyenne, 17 % (± 15 %) des patients provenaient du service des urgences et 52 % (± 24 %) provenaient d’une unité de soins primaires. La plupart des centres avaient accès à des tests spécialisés (prélèvements veineux surrénaliens, études du sommeil de niveau 1, tests autonomes) et à des traitements avancés pour l’hypertension résistante (dénervation rénale). Une hétérogénéité considérable a été constatée en ce qui concerne la pression artérielle cible chez les jeunes présentant un faible risque cardiovasculaire et les algorithmes diagnostiques pour étudier les causes secondaires de l’hypertension.

**Conclusions:**

Ces résultats résument la situation actuelle des soins spécialisés de l’hypertension et font ressortir des occasions d’accroître la collaboration entre les spécialistes de l’hypertension, notamment en ce qui concerne une normalisation de l’approche des soins spécialisés pour les patients hypertendus.

Hypertension, which affects nearly 6 million Canadian adults, is a leading risk factor for stroke, cardiovascular disease, kidney failure, atrial fibrillation, and dementia.^1^^,^^2^ Those at highest risk of developing complications of hypertension include the approximately one third of Canadians whose blood pressure (BP) remains above recommended BP targets, and the 10%-15% who are estimated to have treatment-resistant hypertension are at even greater cardiovascular risk.^3^ These statistics underscore the need for accessible and effective clinical care delivery to optimize control and avoid hypertension-mediated adverse health sequelae.

Provision of specialist hypertension clinical care is an important component of an overall BP control strategy. Hypertension specialists focus on the assessment and management of complex patients. The types of referrals seen by hypertension specialists include the following: resistant hypertension (BP that remains above target despite treatment with 3 antihypertensive medications at optimal doses including a diuretic),^4^; refractory hypertension (uncontrolled BP despite 5 or more antihypertensive agents, including a long-acting thiazide-like diuretic and a mineralocorticoid receptor antagonist),^5^; labile BP; autonomic dysfunction; and diagnosing and managing secondary hypertension. In addition, hypertension specialists often oversee 24-hour ambulatory BP-monitoring programs.

In many cases, evidence to guide care in complex hypertension is limited. Further, contemporary hypertension clinical practice guidelines, focused on diagnosis and initial management, lack evidence for in-depth management of secondary and complex cases of hypertension.^6^, ^7^, ^8^ In these settings, clinical experience is an asset, and access to specialized resources, or lack thereof, may influence clinical decision-making and outcomes.

As little is known about the current state of specialist hypertension practice in Canada, we identified and surveyed hypertension experts in major Canadian hypertension centres, with the aim of better characterizing practice patterns, available resources, populations served, and services provided.

## Methods

### Ethics approval

Ethics approval was obtained from the University of Alberta before initiating study procedures (#Pro00120239). Details regarding informed consent were provided to each potential respondent, and consent was implied by completion of the survey.

### Identifying hypertension specialists

To identify adult hypertension specialists and hypertension centres, we identified the Directors of the Divisions of Endocrinology, Nephrology, General Internal Medicine, and Cardiology at each Canadian University with an affiliated medical school, through an Internet search. We e-mailed the divisional directors, asking them to identify one hypertension specialist (if any), defined as the physician to which complex hypertension referrals were directed, per division, and per major hospital or community clinic site (if more than one site was present). Each divisional director was sent 2 reminders if no response was received.

Identified hypertension experts were then contacted by e-mail and invited to complete an online questionnaire. Each clinician was sent 2 reminders to complete the survey. Participating hypertension experts were invited to be coauthors of this article but were not otherwise offered additional incentives.

### Survey development and dissemination

The survey (Supplemental Appendix S1) was internally created, tested for face validity, and revised by 4 authors (S.L., L.D., R.P., J.R.). The survey was sent in September 2022, through SurveyMonkey, via e-mail to the identified hypertension specialists. One physician of each specialty in each hypertension clinic completed the survey. Survey content included the following information related to the respondents’ practice:

1. Service providers: number, background training, full-time equivalence, and trainee presence;
2. Service provision: referral population, catchment area, referral volume, capacity, and wait time for urgent referrals;
3. Specialized resources and services: availability of 24-hour ambulatory BP monitoring, adrenal vein sampling, hypertension in pregnancy expertise, renal denervation, level 1 & 3 sleep studies, and autonomic testing;
4. Approaches to clinical care: assessed by presenting clinical cases and outlining management for a young patient with hypertension, renovascular hypertension, hyperaldosteronism, pheochromocytoma, and Cushing’s syndrome; and
5. Quality improvement initiatives: local case review, and care quality tracking.

### Statistical analysis

Descriptive statistics, including calculation of mean (standard deviation), median (interquartile range), counts and percentages, were used to collate and report the survey results. Missing data were excluded from the analysis; missing data occurred in up to 6 respondents for some questions.

## Results

**Figure 1:**
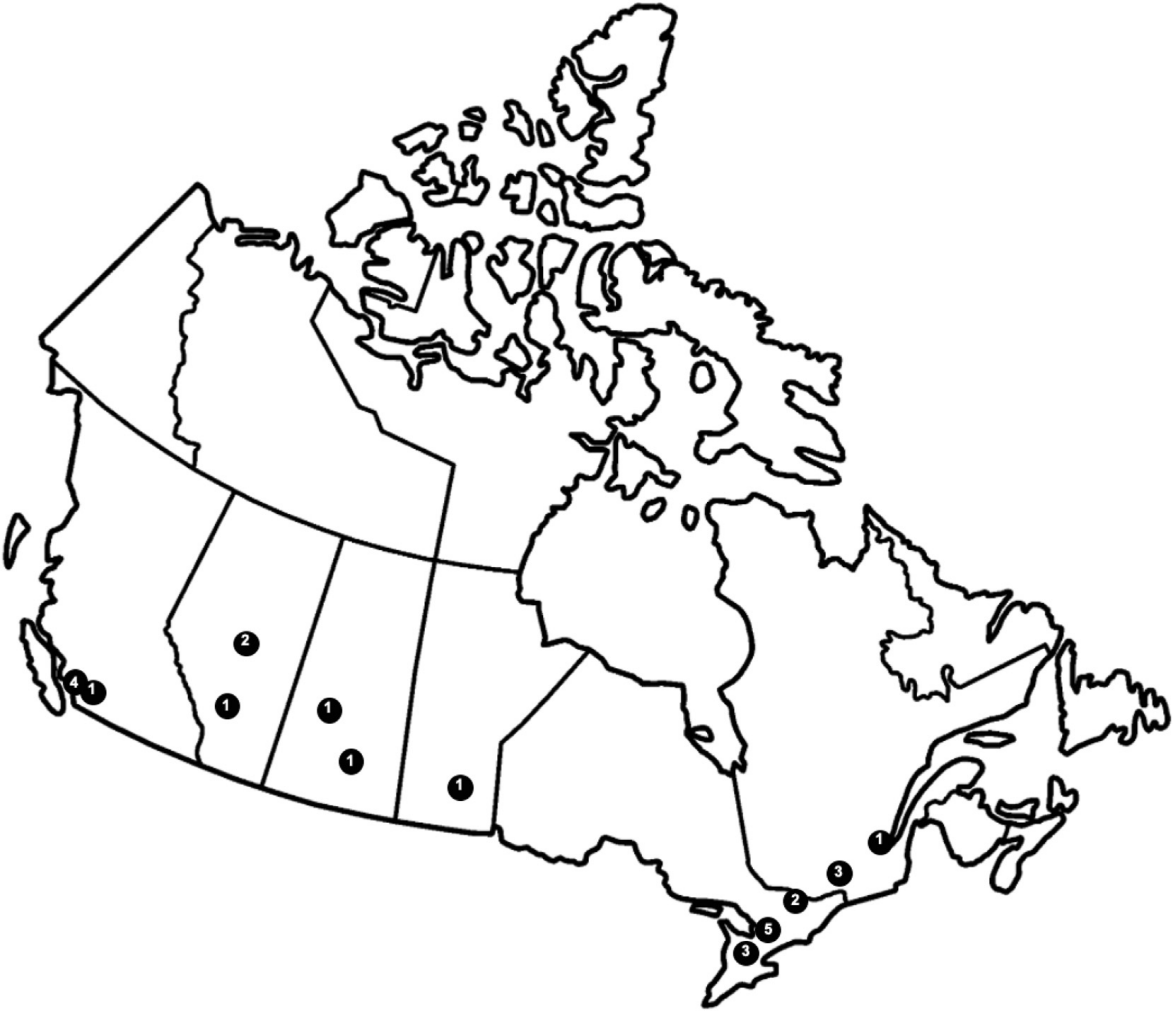
Distribution of Canadian hypertension specialists who responded to our survey.

The initial request for identifying hypertension experts was sent to 63 divisional directors across Canada. Twenty responses were received, and 34 hypertension experts from cardiology, endocrinology, nephrology, and general internal medicine were identified and contacted via e-mail. Twenty-five of 34 (73.5%) completed the survey (Table 1), including respondents from Vancouver (n = 4), Surrey (n = 1), Calgary (n = 1), Edmonton (n = 2), Saskatoon (n = 1), Regina (n = 1), Winnipeg (n = 1), Toronto (n = 5), Hamilton (n = 3), Ottawa (n = 2), Montreal (n = 3), and Quebec City (n = 1) across 25 sites (Fig. 1). Surveys were sent to providers in the Maritimes, but responses were not received. One questionnaire was completed only partially and was excluded from the analysis. Therefore, a total of 25 surveys were included in the analysis.

**Table 1:** Demographics of respondents to hypertension survey

Demographic	Specialists (n = 25)
City of practice	
British Columbia	5 (20)
Alberta	3 (12)
Saskatchewan	2 (8)
Manitoba	1 (4)
Ontario	10 (40)
Quebec	4(16)
Maritimes	0 (0)
Subspecialty of practice	
Cardiology	3 (12)
Nephrology	10 (40)
General internal medicine	8 (32)
Endocrinology	4 (16)

Values are reported as count (%).

The primary subspecialties of respondents included nephrology (n = 10), general internal medicine (n = 8), endocrinology (n = 4), and cardiology (n = 3). The number of half-days per clinic dedicated each week to providing care to patients with hypertension ranged from 1 to 10, with a median of 3.

### Service providers

The number of healthcare providers working per site ranged from 1 to 23 (median 3; interquartile range 1-6). At least one certified hypertension specialist (certification from the American Hypertension Specialist Certification Program, formerly the American Society of Hypertension (ASH) Hypertension Specialists Program) was working in 15 of 25 (60%) of the responding centres. Three (12%) of the surveyed sites were community sites. Many of the hypertension specialist centres (44%) were multidisciplinary and included at least one of the following: hypertension-trained nurses, pharmacists, kinesiologists, registered dieticians, and physiotherapists. Medical trainees, ranging from medical students to senior subspecialty residents, were present at 21 sites (84%). Educational experiences ranged from single clinics to multiple clinics in a block, to a longitudinal experience over months.

### Service provision

Only one centre had Hypertension Centre Certification through the American Heart Association. Each centre was known for expertise in unique areas of hypertension, with some sites specializing in more than one area. Specifics regarding service provision are reported in Table 2.

**Table 2:** Service provision

Clinics with areas of specialization (some clinics have more than one area of specialization)					
	Number of sites (n = 25)	Mean ± SD	Range	Median	IQR
Specialization					
Endocrine hypertension	15				
Renovascular disease	13				
Intrinsic renal disease	10				
Obstetrical medicine	5				
Autonomic dysfunction	6				
Sleep-disordered hypertension	2				
Weight management	5				
Cardiovascular risk reduction	13				
Wait times for initial consultation					
Time, mo					
< 1	4				
1–3	15				
3–6	4				
6–12	2				
Estimated percentage of cases seen by type of hypertension					
Type of hypertension	Number of respondents (n = 25)				
Primary	24	42 ± 25	5-80	45	37.5
Secondary	24	42 ± 25	5-95	30	32.5
Resistant	23	32 ± 22	10-80	25	30
Orthostatic	20	9 ± 7	0-25	5	7.5
Autonomic dysfunction	20	6.1 ± 8	0-35	5	8
Origin of referrals					
Referring provider					
Primary care	25	52 ± 24	10-100	40	30
Emergency department	21	17 ± 15	0-50	10	22.5
Other medicine specialists	23	36 ± 22	5-80	30	30

Values are %, unless otherwise indicated.

IQR, interquartile range; SD, standard deviation.

Approximate wait times for initial consultation ranged from less than 1 month to 12 months; data for wait times are presented in Table 2. Two of the centres with 3-6-month wait times had one physician at the site seeing patients; the remaining sites with longer wait times had multiple physicians seeing patients at the site. Provisions were in place for urgent referrals at 24 of 25 centres. Timelines for urgent referrals are presented in Table 2. Catchment areas for care were also quite variable and ranged from a portion of one city, to half a province, to several provinces.

Overall estimated patient volumes per year, per clinic, ranged from 50 to 2500 patients (24 respondents; mean 738 ± 972; median 425; interquartile range 800). The types of hypertension cases seen varied per site. One respondent did not answer this question, and overall response number was 20-24 depending on the type of hypertension. Results are reported in Table 2.

### Specialized resources and services

Ambulatory BP-monitoring programs were available at 21 of 25 sites (84%), with the number of ambulatory BP-monitoring studies performed ranging from 0 to 2000 studies per year. A self and/or home BP-monitoring software platform for patients was available at 6 sites, with the Sphygmo Home software (mmHg Inc., Edmonton, Alberta) being the most frequently used, at 4 sites.

Adrenal vein sampling was available at 24 of 25 sites; the remaining site had access to adrenal vein sampling at another centre. For adrenal vein sampling, the success rate of sampling on the first try was estimated to be from 5% to 100% (22 respondents; mean 76% ± 24%). Hypertensive disorders of pregnancy were managed at 14 centres (56%); the remaining 11 centres referred these patients to another site. Device-based therapy and experimental treatments such as renal denervation were offered at 8 hypertension centres; 6 other sites made referrals to another centre for these therapies. Access to a level 1 sleep lab was available at 23 sites; those centres that did not have direct access referred patients to respirology for consideration of a level 1 sleep study (performed in a sleep laboratory). Level 3 sleep studies (performed in the patient’s home) were accessible to 19 of the hypertension centres. Autonomic testing was available at 11 hypertension sites, and 6 other sites had access through other means (4 sites through cardiology; 2 sites through neurology).

### Approaches to clinical care

Wide variations in practice were observed in clinical care delivery for primary hypertension and in the workup of secondary hypertension. Responses to clinical cases presented in the survey are provided in Table 3.

**Table 3:** Approaches to clinical care

Survey question	Survey responses							
**30-year old male patient with primary hypertension (work-up done for secondary causes all negative); optimized in terms of health behaviors; ABPM 145/92 mm Hg; no target organ damage; no comorbid medical conditions**								
Would you start anti-hypertensive medications? (n = 25)	Yes (n = 14)	No (n = 11)						
If no, at what blood pressure would you start treatment? (n = 10)	160/100 mm Hg (n = 6)	160/90 mm Hg (n = 2)	ABPM with systolic BP > 155 mm Hg or diastolic BP > 95 mm Hg (n = 1)	Variable (n = 1)				
If yes, what blood pressure target do you treat to? (n = 15)	135/85 mm Hg (n = 6)	140/90 mm Hg (n = 2)	130/80 mm Hg (n = 3)	130/90 mm Hg (n = 1)	160/100 mm Hg (n = 1)	Diastolic BP < 80 mm Hg (n = 1)	Systolic BP < 130 mm Hg (n = 1)	
What guidelines do you use (if any) regarding blood pressure treatment thresholds and targets? (n = 25)	Hypertension Canada (n = 21)	ACC (n = 1)	None (n = 2)	More than one guideline (n = 1)				

**Working up patients for pheochromocytoma**								
Do you work up patients with resistant hypertension for pheochromocytoma? (n = 25)	Yes (n = 17)	No (n = 8)						
Do you work-up asymptomatic patients with early-onset hypertension for pheochromocytoma? (n = 25)	Yes (n = 13)	No (n = 12)						
What initial screening test would you use to screen early-onset hypertension for pheochromocytoma? (check all that apply)	Plasma metanephrines & normetanephrines (n = 6)	Urine metanephrines & normetanephrines (n = 13)	Urine norepinephrine & epinephrine (n = 3)	Plasma norepinephrine & epinephrine (n = 0)				
If biochemical positivity and imaging is required for pheochromocytoma, what is your next step? (check all that apply)	MIBG (n = 16)	FDG-PET (n = 4)	DOTATATE PET (n = 6)					

**26-year old female patient; elevated BMI, no significant family history; hypertension controlled on 2 medications; normal endocrine workup for secondary causes; normal sleep study; normal renal function and electrolytes**								
If you do an ultrasound [for consideration of renal causes of hypertension], do you pursue another test if the ultrasound is normal? (n = 25)	Yes (n = 15)	No (n = 10)						
Do you work-up for renovascular disease? (n = 25)	Yes (n = 17)	No (n = 6)	It depends (n = 2)					
If yes, how do you work-up for renovascular disease?	CTA (n = 6)	MRA (n = 6)	Doppler US (n = 4)	Did not specify(n = 1)				

**50-year old male with hypertension on 3 medications; normokalemia; suppressed renin and elevated aldosterone. Patient is interested in and is a candidate for AVS/Surgery if there is unilateral disease; no adenoma on adrenal CT**								
Does your site pursue confirmatory testing? (n = 25)	Yes (n = 16)	No (n = 8)	Defer to endocrinology (n = 1)					
If your site pursues confirmatory testing, which protocol is used? (n = 17)	Saline suppression test (n = 11)	24-hour urine aldosterone (n = 1)	Adrenal vein sampling with cosyntropin suppression test (n = 1)	Captopril suppression test (n = 1)	Sodium loading (n = 2)	Defer to endocrinology (n = 1)		
If no confirmatory testing or after confirmatory testing, what are the next steps your site would pursue?	Adrenal vein sampling (n = 16)	Surgery consult (n = 1)	Sleep study if BP uncontrolled (n = 1)	None (n = 2)	Refer to endocrinology (n = 2)	Medical treatment with MRA (n = 1)	Salt loading with 24-hour urine aldosterone (n = 1)	Repeat aldosterone-renin ratio (n = 1)
During work-up for primary aldosteronism, what concomitant medications does your site consider acceptable?	All except MRA/beta blocker (n = 2)	CCB (n = 1)	Hydralazine, non-dihydropiridine CCB, alpha blocker (n = 8)	Only alpha blockers (n = 1)	All except MRA (n = 8)	All except ACE/MRA (n = 2)	All if renin suppressed (n = 1)	It depends (n = 1)
If you pursue a washout of medications to work-up primary aldosteronism, how long is this pursued before confirmatory testing is done?	1 wk (n = 2)	2 wk (n = 3)	3 wk (n = 3)	6 wk (n = 2)	8 wk (n = 1)	Variable (n = 12)	Not done (n = 1)	

**Working up patients for Cushing’s disease**								
If you are seeing a patient without a known adenoma, do you screen for Cushing’s? (n = 25)	Yes (n = 18)	No (n = 7)						
If you screen for Cushing’s, what test do you use? (n = 18)	Dexamethasone suppression testing (n = 8)	24-h urine cortisol (n = 7)	AM cortisol (n = 3)					
Is there variability at your site with respect to screening for Cushing’s? (n = 18)	Yes (n = 12)	No (n = 6)						

ABPM, ambulatory blood pressure monitoring; ACC, American College of Cardiology; ACE, angiotensin-converting enzyme; AM, morning; AVS, adrenal vein sampling; BMI, body mass index; BP, blood pressure; CCB, calcium-channel blocker; CT, computed tomography; CTA, computed tomography angiography; DOTATATE PET, dotatate positron emission tomography; FDG-PET, fluorodeoxyglucose-positron emission tomography; MIBG, metaiodobenzylguanidine; MRA, mineralocorticoid receptor antagonist; US, ultrasound.

### Quality improvement initiatives

All respondents completed questions about quality improvement. Nine respondents reported dedicated rounds to discuss hypertension topics and cases, with 6 sites convening rounds monthly, and 3 centres convening rounds yearly. Three centres tracked quality indicators on a systematic basis. BP control rates, medication adherence, adrenal vein sampling success, and long-term response to surgery are examples of the quality indicators tracked.

## Discussion

To our knowledge, this study is the first to examine practices of hypertension specialists across Canada. This survey provides a cross-section of specialist hypertension care in Canada, covering many hypertension centres.

These survey results demonstrate significant variability in hypertension specialist clinical practice views in Canada. This finding is not surprising given the lack of high-quality data in patients with complex hypertension and secondary hypertension and the variability in recommendations in clinical practice guidelines.

Our findings are similar to those of previous surveys assessing physician attitudes and practices in hypertension in other countries. A previous survey of cardiologists, internists, and general practitioners in the US studying pharmacologic treatment of hypertension with clinical cases found that despite most respondents being in agreement with established hypertension guidelines from the Joint National Committee, significant differences were present across specialties in choosing drug classes to treat hypertension, with cardiologists being more likely to choose angiotensin-converting enzyme inhibitors and calcium-channel blockers compared to internists and family physicians.^9^ Our survey is too small to determine whether the type of subspecialty training has a significant impact on practice patterns among hypertension specialists in Canada.

Another cross-sectional questionnaire surveying resident physicians in internal medicine, family medicine, and surgical specialties in the US about treatment of inpatient hypertension also found that although the majority of trainees based their management on Joint National Committee hypertension guidelines; respondents were divided on how they chose to manage asymptomatic, moderately elevated BP as an inpatient, with 44% starting an antihypertensive medication, and 56% deciding not to initiate pharmacologic treatment.^10^ Although both studies focused on primary hypertension, they highlight the discrepancies between treatment guidelines and clinical practice, which were seen in our study also. We expected such discrepancies, specifically for complex cases of hypertension including secondary and resistant hypertension, as no consensus on management has been reached.

Potential reasons for practice pattern variation were not specifically addressed in our survey. A dearth of information is available in the literature for many of the clinical scenarios addressed in our survey, and for other scenarios, the literature presents variability. For example, 56% of respondents treat young patients with uncomplicated stage 1 hypertension with pharmacologic therapy once health behavior is optimized, whereas the others would wait for higher BP readings. This variability in practice is reflected in the variability among hypertension guidelines. For example, the 2017 American College of Cardiology and American Heart Association hypertension guidelines, and the 2018 European Society of Cardiology and European Society of Hypertension guidelines both suggest treatment if BP ≥ 140/90 mm Hg in all adult patients.^8^^,^^11^ However, in patients without cardiovascular risk factors, Hypertension Canada recommends initiating treatment if BP ≥ 160/100 mm Hg.^6^ The number needed to treat for this average-risk population is high, which explains the higher Hypertension Canada threshold, whereas other guidelines offer simplified thresholds and targets.^12^

Specialists in this study reported seeing patients with primary hypertension, secondary hypertension, resistant hypertension, orthostatic hypotension, and autonomic dysfunction. Many of these conditions are common. For example, resistant hypertension is estimated to affect 10% of treated patients with hypertension in the primary care setting.^13^ Using traditional biochemical criteria for primary aldosteronism (PA), prevalence has been reported at 4.8%-16.6%, but with a more liberal biochemical definition, the prevalence could be as high as 13.8%-24.5%.^14^ Although this level of prevalence is reported for PA, a Canadian study found that the detection and treatment of expected PA cases is less than 1%.^15^ This study suggests that a system-level approach to assist with investigation and treatment of PA could be effective in closing gaps in care and improving clinical outcomes.^15^ Based on the estimated prevalence of these conditions, most patients with complex and secondary causes of hypertension are likely to be managed exclusively by primary care and by other specialist colleagues, rather than in specialized hypertension centres.

Another unknown is what factors influence referral patterns to hypertension specialist clinics, and which patients are best served in these clinics. The availability and wait times for hypertension specialist care vary across the country, as do resources, including allied-health professional availability. Given the well described disparities in hypertension control rates by sex, race, ethnicity, and other socioeconomic factors,^16^^,^^17^ an important consideration is whether access to hypertension specialist care in Canada is equitable. Furthermore, a quarter million Canadians have resistant hypertension in Canada^3^; consideration of how to meet the needs of this growing population given the existing clinic volumes reported in this survey will be needed. In internal medicine residency programs, ambulatory care exposure is limited,^18^ which is where most hypertension care occurs. Heterogeneity in training experiences may explain some of the heterogeneity in practice patterns. More work is needed to better define whether patients are getting the right care at the right time delivered by the right provider.

This study also highlights opportunity for future work to describe hypertension control rates at each centre, choice of pharmacotherapy at each centre, and factors influencing the choice. We also found some survey responses that were unusual (Table 3) and warrant follow-up questions; perhaps more clarity is needed in the survey questions. This survey did not have the option of text to explain the answers provided. Given that the majority of hypertension care occurs in primary care, further surveys of primary care providers on the provision of hypertension care would augment this work.

To improve and standardize care delivery nationally, survey participants indicated interest in having case conferences on a variety of topics, including causes of secondary hypertension (renovascular disease, primary aldosteronism), autonomic dysfunction and orthostatic hypotension, and medical and interventional approaches to treatment-resistant hypertension. The Canadian Hypertension Specialists Society (CHeSS) recently created a virtual forum for these presentations. Survey participants also proposed topics for standardized protocols and evidence-based summaries on secondary causes of hypertension. The results of this survey, the renewed energy for ongoing learning by responding clinicians, and the recently published worsening hypertension control rates in Canada between 2007 and 2017^1^ are impetus to continue efforts to raise awareness of hypertension, improve clinician education, and improve both access to care and hypertension control rates in Canada.

Several limitations of our study need to be considered. We were unable to identify a hypertension expert at every medical school–affiliated Canadian university, which may reflect the lack of hypertension specialist availability and/or be related to our method of identifying specialists. Our method of identifying experts through academic centres may miss those specialists who are not known to the divisional directors, including those practicing in the Yukon, Northwest Territories, and Nunavut, where no specialists were identified. Our e-mails were sent in English, which may have limited the responses provided by non–English speaking hypertension experts.

An alternate sampling method of reaching out to only to physicians certified through the American Hypertension Specialist Certification Program may miss those specialists who do not have this certification. In addition, clinical case scenario responses represent the practice pattern of the individual responding on behalf of the centre and may not represent the practice patterns of all healthcare providers at each centre. Furthermore, respondents were asked to estimate the percentages reported here; thus, they may not be a true representation of the respondent’s practice.

## Conclusion

In this survey of hypertension specialists across Canada, we found significant variability in practice patterns. We found a keen interest among clinicians in sharing ideas and case discussions and the desire to distill ideas and create guidance statements and position papers in clinical situations for which little guidance is available beyond expert opinion.
