# Supplementary material for: Characterizing Hypertension Specialist Care in Canada: A National Survey
Source: CJC Open. 2023 Sep 10;5(12):907–15. doi: 10.1016/j.cjco.2023.08.014 (PMC10774075; doi:10.1016/j.cjco.2023.08.014)
Supplement: Supplemental Appendix S1 [file mmc1.pdf]

SUPPLEMENTARY MATERIAL

Supplemental Appendix S1. Hypertension Survey

National Survey of Hypertension Centers:

Please complete these questions on behalf of all personnel involved in hypertension care in your center.

Medical Personnel:

---

1. **How many health care providers (physicians, nurses, pharmacists, kinesiologists, registered dieticians, etc.) work in your Hypertension Center?**

   a. *Please list the health providers (with FTE) that participate in your clinic, their role, whether they have direct patient interaction, how many of the total referred patients they see and their qualifications specific to hypertension.*

      Provider 1:________
      Role________
      FTE_______
      Direct patient interaction?_______
      Percentage of total referred patients seen:__________
      Qualifications specific to hypertension:________

      Provider 2:________
      Role________
      FTE_______
      Direct patient interaction?_______
      Percentage of total referred patients seen:__________
      Qualifications specific to hypertension:________

      Provider 3:________
      Role________
      FTE_______
      Direct patient interaction?_______
      Percentage of total referred patients seen:__________
      Qualifications specific to hypertension:________

      Provider 4:________
      Role________

FTE_______
Direct patient interaction?_______
Percentage of total referred patients seen:__________
Qualifications specific to hypertension:________

Provider 5:________
Role________
FTE_______
Direct patient interaction?_______
Percentage of total referred patients seen:__________
Qualifications specific to hypertension:________

Provider 6:________
Role________
FTE_______
Direct patient interaction?_______
Percentage of total referred patients seen:__________
Qualifications specific to hypertension:________

2. **How many half days per week are patients seen in your hypertension clinic? _______**

3. **Are any of your providers ASH certified?**

   Yes___ No___

   If Yes:
   How many providers are ASH-certified? _______

4. **Please describe the MD background specialty (IM/GIM/Cardio/Neph/Endo) of the attending physicians in your clinic?**

   Physician 1:_____
   Physician 2:_____
   Physician 3:_____
   Physician 4:_____
   Physician 5:_____

5. **Are there trainees in the clinic?**

   Yes____ No____

   If Yes:
   a. What level are the trainees and how many per year would rotate through:
      UGME:____

PGME R1-3:_____ Home program(s) (i.e: Core internal medicine, surgery, obstetrics and gynecology):__________
PGME R4-6:_____ Home program(s) (i.e: Endocrinology, Nephrology, GIM, IM):__________

b. Do the trainees spend longitudinal time in the clinic or parachute in for single clinics (see patients only once)?
% with longitudinal experience:_______
% with single clinic experience:_______

Service Provided

1. **Is your center ASH certified?**

   Yes____ No____

2. **What is the catchment area for which the center provides care for patients with hypertension?** ________

3. **Is there an ambulatory blood pressure monitoring program at your center?**

   Yes____ No____

   a. How many ABPM studies are done each year:________
   b. Who interprets the ABPM studies:__________________
   c. Where was their expertise obtained: __________________

4. **Is there a self or home BP telemonitoring platform provided for patients at your center?**

   Yes____ No____

   If Yes,
   Which one:______________

5. **What are the approximate wait times for patients to come see a hypertension specialist at your center?**

   Less than 1 month_______
   1-3 months________
   3-6 months_________

6-12 months________
Greater than 12 months_________

6. **Are there provisions for urgent referrals?**

   Yes____ No____

   a. What is the timeline for an urgent referral?

   Less than 1 week_____
   1-2 weeks_________
   2-4 weeks_________
   >4 weeks__________

7. **Is your center known for a particular area of hypertension? (Please check all that apply)**

   Endocrine HTN (PA, pheochromocytoma, Cushing's)______
   Renovascular (FMD, atherosclerotic)______
   Intrinsic Renal (PCKD, glomerular nephritis, CKD)____
   Obstetrical Medicine_____
   Autonomic dysfunction_____
   Sleep disorders_____
   Weight management____
   CV risk reduction______

Referral Population

---

1. **How many patients per year are seen in your center:**_______

2. **What is the estimated percentage of each of the following seen in your clinic:**
   Primary hypertension:_____
   Secondary hypertension:_______
   Resistant hypertension: ________
   Orthostatic hypotension:________
   Autonomic dysfunction:_________
   Other (please specify):____________

3. **Where do your referrals originate (percentage for each):**
   Primary care:_______
   Emergency Department:_______
   Other medicine specialists:________

Other (please specify):__________

Specialized Resources

1. **Does your center have access to adrenal vein sampling?**

   Yes____ No____

   If No:
   a. Is there another center where you have access to adrenal vein sampling?

      Yes____ No____

   If Yes:
   b. When you refer patients for adrenal vein sampling, what is the success rate (%) of accurate samples on first try_______

2. **Does your center see women with hypertensive disorders of pregnancy?**

   Yes____ No____

   If No:
   a. Is there another center where patients in your area have access to expert care in hypertensive disorders of pregnancy?

      Yes____ No____

3. **Does your center offer device-based therapy and experimental treatments like renal denervation?**

   Yes____ No____

   If No:
   a. Is there another center where you might refer patients for device-based therapies?

      Yes____ No____

4. **Does your center use or have access to a level 1 sleep lab?**

   Yes____ No____

   If No:
   a. Do you have access through respirology or other means?___

5. **Does your center have access to level 3 sleep studies?**

Yes____ No____

If No:
a. Do you have access through respirology or other means?____

6. **Does your center use or have access to autonomic testing?**

Yes____ No____

If No:
a. Do you have access through another specialty?

Yes_____through______ No____

Specific Cases

Please outline your center's approach to the following:

30M with primary hypertension (work up done for secondary causes, all negative), optimized in terms of health behaviors, ABPM 145/92. No target end organ damage, no co-morbid medical conditions.

Do you start anti-hypertensive medications?

Yes____ No____

If Yes:
a. What blood pressure target do you treat to? ______

If No:
b. At what blood pressure would you start treatment? ______

What guideline do you use (if any) regarding BP treatment thresholds and targets? ______

Do you work up asymptomatic patients with resistant hypertension for pheochromocytoma?

Yes____ No____

Do you work up asymptomatic patients with early-onset hypertension for pheochromocytoma?

Yes____ No____

If Yes:

a. With what initial screening test? (check all that apply)
   Plasma metanephrines and normetanephrines_____
   Plasma norepinephrine and epinephrine_____
   Urine metanephrines and normetanephrines_____
   Urine norepinephrine and epinephrine_____
   Other (please specify): _____

If biochemical positivity and imaging required, what is your next step?

MIBG_____
FDG PET_____
DOTATATE PET_____

26F woman, overweight, no significant family hx, hypertension controlled on 2 medications, normal endocrine workup for secondary causes, normal sleep study, normal renal function and electrolytes.
Do you/how do you work up for renovascular dx? (Doppler US vs CTA vs MRA vs no investigations)

If you do an ultrasound, do you pursue another test if the ultrasound is normal?
Yes____ No____

50M with hypertension on 3 medications, normokalemia, suppressed renin and elevated aldosterone. Patient interested in and candidate for AVS/Surgery if unilateral disease. No adenoma on adrenal CT.
Does your site pursue confirmatory testing? Yes____ No____
If so, which protocol?_________
If no confirmatory testing or after confirmatory testing, what are the next steps your site would pursue:___________.

During workup for PA, what concomitant medications does your site consider acceptable: ____.

If you pursue a washout of medications, how long is this pursued before confirmatory testing is done: ___________.

If you are seeing a patient without a known adenoma, do you screen for Cushing's? Yes____ No____
If so, with what test?
Is there variability at your site with respect to screening for Cushing's? Yes____ No____

If Yes:

a. How much estimated variability is there?

1. **At your center, are there rounds to discuss hypertension topics and cases?**

   Yes____ No____

   If Yes:
   b. How often do these occur?
      Weekly_____
      Monthly_____
      Yearly______

2. **At your center do you track any quality indicators on a systematic basis?**

   Yes____ No____

   If Yes:
   a. Which indicators does your center use:
      BP control rates____
      Medication adherence____
      AVS success____
      Long-term response to surgery____

CHESS Survey

---

1. **Are there particular topics you would like covered in the CHESS case conferences? Please list them below and offer suggested speakers:**_________________________

2. **Do you have a case you would like to discuss? Please outline the topic:**_______________

3. **Are there specific protocols you would like to see CHESS develop?**

4. **What publications would you like to see originate from CHESS?**
